# Tackling the global plastic pollution crisis

**DOI:** 10.1093/nsr/nwag582

**Published:** 2026-09-08

**Authors:** Guibing Zhu, Changchao Li, Peng Wang, Peixiu Chen, Jingjing Peng, Ziyang Tang, Lei Zhong, Chunlei Liu, Ze Ding, Shanyun Wang, Nan Zhang, Liping Jiang, Hans Peter H Arp, Matthias C Rillig, Yong-Guan Zhu

**Affiliations:** Research Center for Eco-Environmental Sciences, Chinese Academy of Sciences, Beijing 100085, China; University of Chinese Academy of Sciences, Beijing 100049, China; Department of Civil and Environmental Engineering, The Hong Kong Polytechnic University, Hong Kong, China; State Key Laboratory for Ecological Security of Regions and Cities, Institute of Urban Environment, Chinese Academy of Sciences, Xiamen 361021, China; School of Fashion and Textiles, The Hong Kong Polytechnic University, Hong Kong, China; College of Resources and Environmental Sciences, China Agricultural University, Beijing 100193, China; Research Center for Eco-Environmental Sciences, Chinese Academy of Sciences, Beijing 100085, China; University of Chinese Academy of Sciences, Beijing 100049, China; School of Earth System Science, Tianjin University, Tianjin 300072, China; Research Center for Eco-Environmental Sciences, Chinese Academy of Sciences, Beijing 100085, China; University of Chinese Academy of Sciences, Beijing 100049, China; Research Center for Eco-Environmental Sciences, Chinese Academy of Sciences, Beijing 100085, China; Research Center for Eco-Environmental Sciences, Chinese Academy of Sciences, Beijing 100085, China; University of Chinese Academy of Sciences, Beijing 100049, China; Research Center for Eco-Environmental Sciences, Chinese Academy of Sciences, Beijing 100085, China; University of Chinese Academy of Sciences, Beijing 100049, China; Research Center for Eco-Environmental Sciences, Chinese Academy of Sciences, Beijing 100085, China; Norwegian Geotechnical Institute (NGI), Oslo NO-0806, Norway; Department of Chemistry, Norwegian University of Science and Technology (NTNU), Trondheim NO-7491, Norway; Institute of Biology, Freie Universität Berlin, Berlin 14195, Germany; Berlin-Brandenburg Institute of Advanced Biodiversity Research, Berlin 14195, Germany; Research Center for Eco-Environmental Sciences, Chinese Academy of Sciences, Beijing 100085, China; University of Chinese Academy of Sciences, Beijing 100049, China

**Keywords:** plastic pollution, microplastics, nanoplastics, environmental impacts, management

## Abstract

Plastic pollution has emerged as a critical global environmental challenge, exacerbating the impacts of climate change and biodiversity loss while posing substantial risks to human health and the economy. Despite a rapid increase in scientific efforts in recent years, our understanding of the characteristics, distribution and environmental consequences of plastic pollution remains incomplete, with many aspects still under debate. This knowledge gap hinders the development and implementation of effective management strategies. In this Review, we provide a comprehensive synthesis of plastic pollution from understanding it to combatting it, linking the environmental behavior of plastics with their ecological, health and Earth-system risks. We further discuss mitigation and management strategies across the plastics life cycle. These strategies emphasize upstream prevention through redesign for minimizing production and facilitating reuse, supplemented by measures such as recycling and the implementation of biodegradable and compostable plastics, as well as exposure risk management. We also identify key challenges to address the interlinked triple crises of climate change, biodiversity loss and pollution associated with plastics. The priorities and solutions to end plastic pollution include promoting sustainable production and consumption, minimizing plastic waste and leakage, mitigating plastic pollution and damage, and safeguarding planetary health.

## Teaser

This Review synthesizes current knowledge of the occurrence, fate, and impacts of plastic pollution, and discusses strategies and priorities for its prevention, mitigation, and management across the plastics life cycle.

## INTRODUCTION

Plastic production has flourished since its first use in consumer products in the 1950s, with plastic production now exceeding 450 million tons per year [1]. Due to a lack of effective management, the majority (∼80%) of plastic waste continues to accumulate on Earth [2,3], presenting a threat to planetary health [4–6]. It is estimated that, by 2050, ∼12 billion tons of plastic waste will have accumulated in natural environments and landfills globally [2], some of which near the sea level may be eroded due to climate change and sea level rise [7]. In the ocean, plastics are transported by currents and can accumulate in biodiverse areas, including gyres, coral reefs and the seabed; plastics can also impact benthic ecosystems in riverbeds and the deep ocean [8–11]. Through this accumulation, plastic waste has exacerbated the interlinked triple planetary crisis of climate change, biodiversity loss and pollution [6,12,13].

While plastic contamination is ubiquitous, there remains a significant gap in understanding the extent and impact of plastic leakage, as well as the primary pathways through which humans encounter plastics, such as through ingestion or inhalation [14]. Microplastics, generally defined as plastic particles <5 mm, and nanoplastics, often defined as plastic particles <1 μm, have become recognized as pervasive environmental pollutants [15]; however, much about their behavior and effects remains poorly understood, particularly in their relation to being a carrier for hazardous substances to various organs [6,16–18]. Recent advances in detection and analytical techniques for plastics have been notable, yet further research is needed to explore the complex

interactions between plastics and additives. While much of the current literature focuses on marine environments and virgin plastics, research has increasingly expanded to include soil and freshwater ecosystems, which are also heavily impacted by plastic pollution [19–23]. Impacts of plastic contamination on various ecosystems and human health are still being evaluated from multiple perspectives [14,15,21,22,24,25].

Addressing the challenge of plastic pollution requires clarifying some fundamental issues. An important issue arises from the overproduction and excessive use of plastics beyond what is necessary or essential, particularly for short-lived applications [26,27]. Second, while the chemical stability of plastics affords the potential for recycling, this capacity has not yet been effectively harnessed; current systems for plastic reuse and recycling remain markedly inefficient [27,28], largely because of the complex chemical composition giving recyclate unknown properties and hazards, in addition to logistical considerations [29]. Third, there remains a significant lack of public awareness regarding the harm caused by plastic pollution, and the issue has not received the attention or prompted the behavioral changes necessary to mitigate its impact [30,31].

This Review aims to provide a comprehensive synthesis of plastic pollution from understanding it to combatting it. We first summarize how plastics are produced, released, transported, transformed and accumulated across environmental compartments. We then examine how these processes translate into ecological, health and Earth-system risks through the interacting physical, chemical and biological properties of plastics. Building on this understanding, we discuss mitigation and management strategies across the plastics life cycle, from upstream prevention and material redesign to recycling, end-of-pipe control, exposure reduction and global governance. By connecting scientific understanding with practical strategies, this Review provides an integrated basis for future research, policy development and coordinated action to end plastic pollution.

## KNOWLEDGE LANDSCAPE OF PLASTIC POLLUTION

To characterize the current knowledge landscape, we constructed a semantic knowledge graph and a thematic distribution map to reveal relationships among literature themes, visualize their relative prominence and identify critical knowledge gaps (Fig. 1). The knowledge landscape is clustered around research topics related to ‘occurrence and fate’, ‘pollution impacts’, ‘life-cycle mitigation’ and ‘barriers and next steps’. These graphs reveal a closely interconnected research landscape on these four topics, with current studies concentrated on the life-cycle aspects of plastic and plastic pollutants, encompassing the continuous chain from upstream prevention and alternative plastics to widespread occurrence, environmental fate and transport, transformation, recycling, end-of-pipe control, and the resulting environmental and health impacts. Although ecosystem and human exposure are critical to understanding the risks of plastic pollution, current research remains limited regarding exposure pathways, bioaccumulation processes and long-term toxicological effects. Addressing these gaps will be essential for improving risk assessment and providing evidence for governance decisions. Pollution control also represents a major research focus, and existing studies have established a life-cycle management framework covering plastic production, use, recycling, substitution and pollution reduction. In recent years, recycling and reuse, as well as biobased and degradable materials, have become important directions in plastic pollution control research. Beyond the plastic life cycle, research on end-of-pipe removal technologies for plastics in the environment and wastewater infrastructure is expanding, alongside discussion of the limits of this management approach.

**Figure 1.**
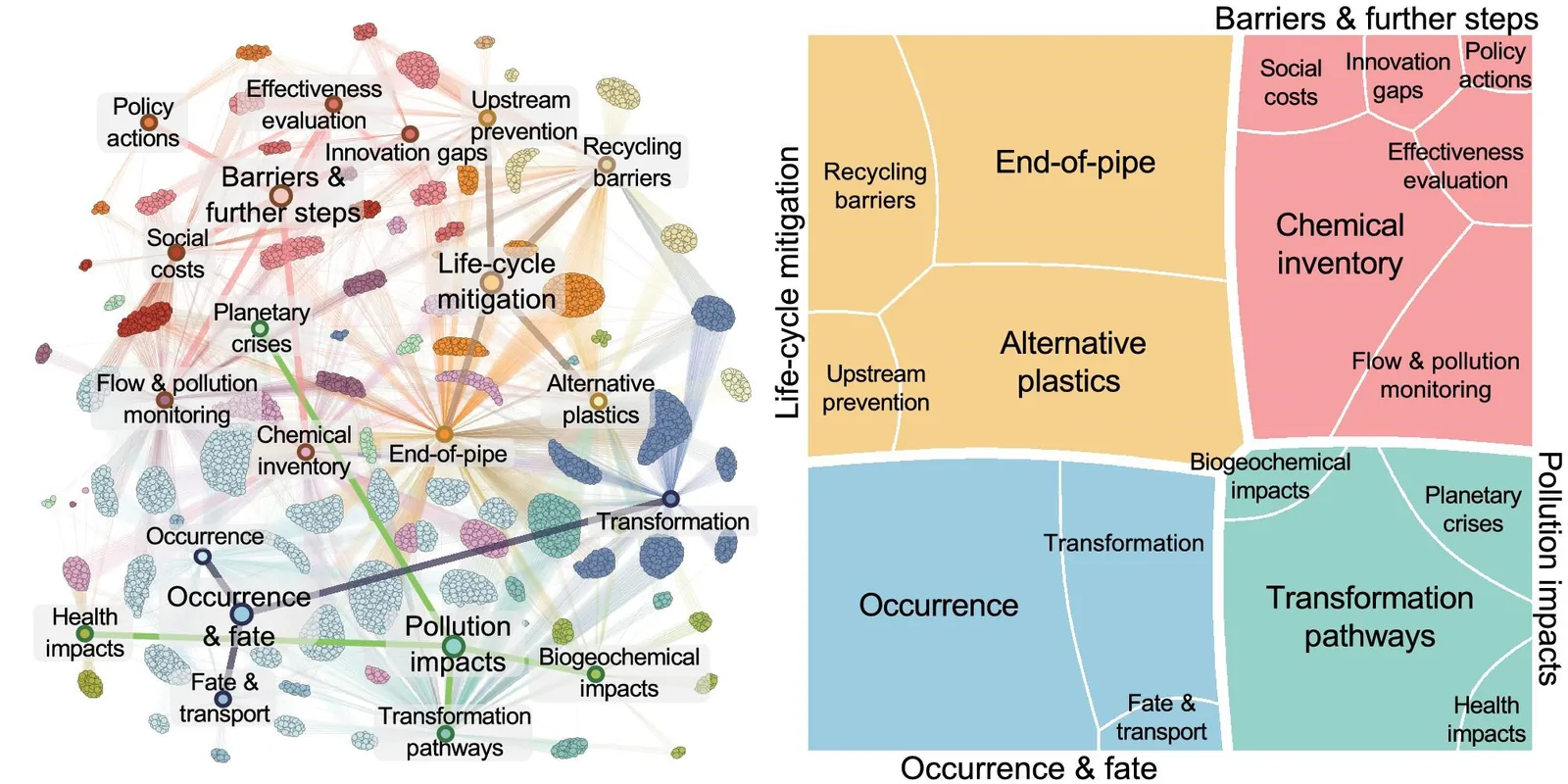
Semantic knowledge graph and thematic distribution map of plastic pollution research (up to February 2026). Data were retrieved from the Web of Science Core Collection using the query ‘plastic OR microplastic OR nanoplastic’ filtered by the category ‘Environmental Sciences’, yielding a total of 36 023 publications. Articles were classified using a sentence-transformer model based on semantic similarity to the keywords of this review’s chapters. The figure on the left depicts the semantic knowledge graph. Each small node represents a cluster of five publications within the same theme, while the larger labeled nodes indicate different themes. The connecting lines illustrate the thematic associations between literature clusters and these labeled themes. The figure on the right shows a thematic distribution map generated by semantic model classification, where the area of each polygon corresponds to the relative volume of each research theme.

Current research still faces major challenges related to understanding the scope of plastic pollution and managing it. In particular, limited monitoring of plastic pollution in the environment makes it difficult to assess whether current policies are effective. Moreover, the transition of emerging technologies from laboratory innovation to large-scale application is constrained by technological maturity, economic feasibility and uncertainties in social costs. As discussed below, further analysis of links among ‘occurrence and fate’, ‘pollution impacts’, ‘life-cycle mitigation’ and ‘barriers and next steps’ can highlight opportunities for interdisciplinary integration among basic science, engineering technologies, policy evaluation and socioeconomic analysis.

## WIDESPREAD OCCURRENCE, FATE, TRANSPORT AND TRANSFORMATION OF PLASTICS

Plastic waste has become ubiquitous across the Earth system, spanning terrestrial, aquatic and atmospheric compartments, from densely populated regions to remote environments such as the polar regions and deep-sea trenches [6,32–34]. Its pervasive occurrence reflects the combined effects of large-scale production, inefficient waste management and long-range transport. Plastic pollutants have been detected in environmental media closely related to human activities and daily life, and continuous releases have increased the likelihood of exposure to organisms. To date, plastic particles have been reported in >1300 species, including fish, mammals, birds and insects [33].

The rapid growth of plastic production has created a large and persistent pool of plastic waste, much of which has accumulated in landfills or the natural environment [2]. Even under ambitious waste-reduction scenarios, plastic pollution is expected to persist because existing polymers are durable and environmental stocks turn over slowly [35]. Climate change may further enhance plastic leakage and remobilization through flooding, landfill erosion and intensified weathering [7,36,37]. These processes can increase the movement of plastics across environmental compartments [36,37]. In addition, weathering and degradation processes can release plastic-associated chemicals and transformation products [36,37]. Biofilm colonization may also alter particle density, surface properties, settling behavior and degradation potential [38–40]. Together, these processes influence the environmental fate and mobility of plastics.

Once released, plastics undergo complex transport and redistribution across compartments (Fig. 2). In aquatic systems, plastics are mobilized by rivers, stormwater runoff, coastal currents and ocean circulation, leading to accumulation in beaches, estuaries, gyres, sediments and deep-sea environments [8,41,42]. On land, plastics can be redistributed through surface runoff, wind erosion, tillage and biotic transport, allowing them to migrate from points of release to soils, groundwater and adjacent ecosystems [42,43]. Because transport is governed by polymer type, particle size, density, shape, weathering state and biofouling, plastic fate varies greatly among materials and environments.

**Figure 2.**
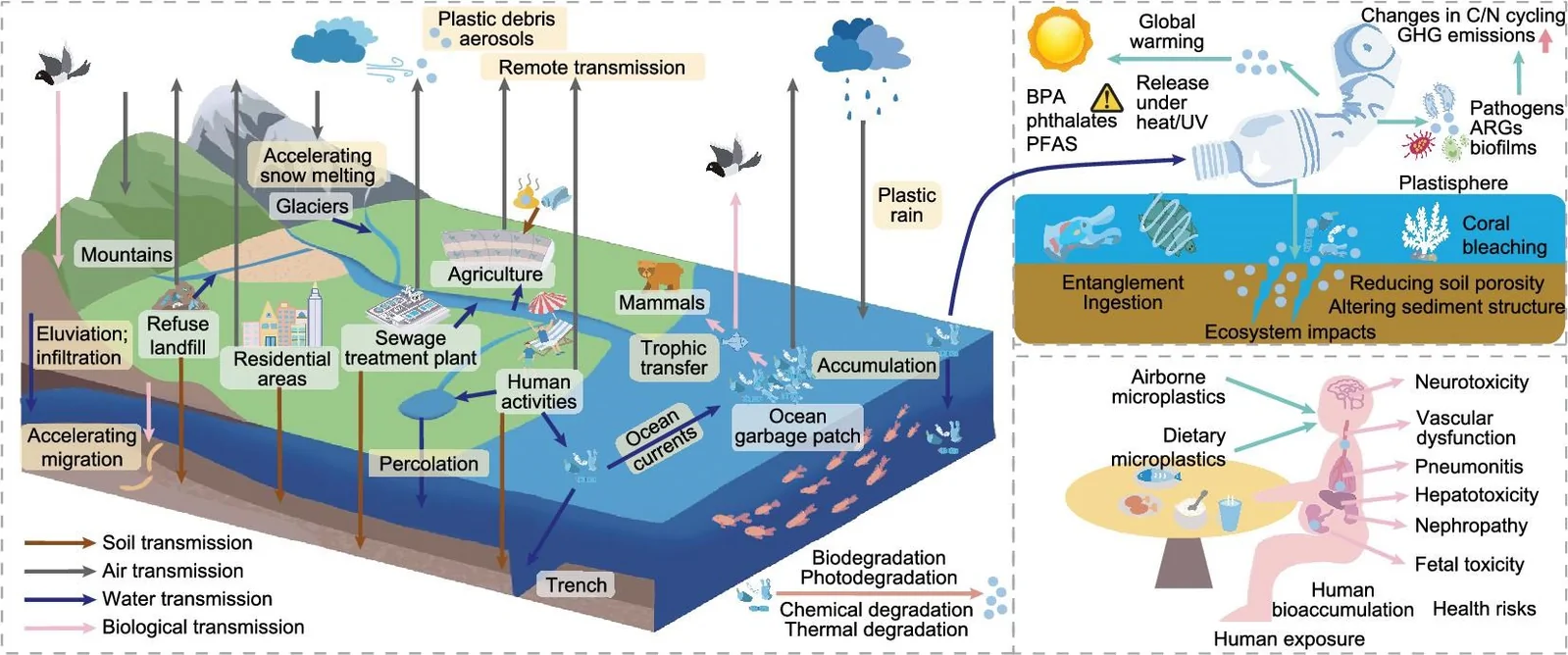
Migration and accumulation pathways of plastics in different environments and potential impacts on ecosystems and human health. The left side illustrates how plastic pollution originates and spreads in terrestrial environments, with sources including landfills, urban activities and agricultural plastics. These plastics disperse into nearby ecosystems via soil leaching, groundwater infiltration and surface runoff, while atmospheric currents and biological carriers (e.g. birds) can transport plastic debris to remote regions. The right panel of the figure spotlights the diverse effects of plastics on ecosystems and human health. Plastics in the environment slowly and continuously release their intrinsic substances such as bisphenol A (BPA), phthalates and per- and polyfluoroalkyl substances (PFAS). The surfaces of plastics can be colonized by microorganisms, including pathogens and antibiotic-resistant bacteria. These processes may disrupt carbon and nitrogen cycles and contribute to broader ecological imbalances. At the same time, in marine environments, plastic litter threatens aquatic species with entanglement and ingestion, contributing to trophic transfer and coral bleaching. In soils and sediments, microplastics can alter soil structure. These pollutants reach the human body through inhalation and dietary intake, with various health risks.

Over time, plastics fragment into microplastics and nanoplastics through physical abrasion, photo-oxidation, thermal stress and microbial action [12,16,44]. These smaller particles are more mobile and can be transported over long distances by water and wind. Accordingly, microplastics have been detected in oceans, freshwater systems, drinking water, shellfish, breast milk, air, mist and rainfall [33,45]. They have also been reported from remote regions such as the Arctic, Antarctic, Mount Everest and the Mariana Trench [6,44,46], indicating that atmospheric transport and long-range dispersal are important pathways in the global plastic cycle [6,47,48]. These observations demonstrate that plastics have become globally distributed contaminants, with complex transport pathways and transformation dynamics across the Earth system (Fig. 2).

## IMPACTS OF PLASTIC POLLUTION

### Physical, chemical and microbial pathways of plastic pollution impacts

The impact of plastic pollution is not limited to the plastics themselves. Chemicals and microbes associated with plastics can also have a profound influence on ecosystems, environments, human health and biogeochemical cycles [6,17,18,21,24,49,50]. Accordingly, an accurate assessment of the real-world consequences of plastic pollution requires comprehensively considering its physical, chemical and microbiological dimensions [18].

#### Physical pathways: habitat modification, ingestion and entanglement

Plastic accumulation on Earth surfaces changes ecosystem landscapes and structures, affecting associated ecological processes and the availability and connectivity of animal habitats. Plastic debris also can affect soil and aquatic environments by changing their physicochemical properties. For example, plastic debris entering soil can change soil particle aggregates, porosity and water holding capacity [51]. This impacts the ecological function of soil ecosystems [4,22,51]. Similarly, in aquatic systems, plastics can influence sediment structure and flow dynamics. Animals often intentionally or unintentionally, or directly or indirectly, ingest plastic debris, blocking their digestive systems [52]. This can lead to malnutrition, reduced growth and ultimately premature death [53]. Large plastic items, such as fishing nets and plastic bags, entangle marine and terrestrial animals [54]. This leads to injuries, restricted movement or suffocation in marine species [55].

#### Chemical pathways: release and transport of plastic-associated chemicals

There are over 16 000 chemicals related to plastics and their production [17]. Of these, over 4200 are recognized as chemicals of concern because of their persistent, bioaccumulative, mobile or toxic properties [17,50]. Plastic-associated chemical pollution is therefore both widespread and persistent. For example, per- and polyfluoroalkyl substances (PFAS) can persist in the environment and may require centuries or millennia to degrade [56]. These chemicals include residual monomers/ligands generated during polymerization; compounds generated during polymer degradation; and additives such as lubricants, antioxidants, plasticizers, colorants and ultraviolet (UV)/thermal stabilizers [17]. These harmful chemicals that are produced and emitted during the plastics life cycle may collectively pose a threat to both humans and ecosystems [57].

Additives can increase the longevity, performance and functionality of plastics; however, plastics can gradually release these compounds into the environment over time. Some additives have been identified as environmental pollutants, including phthalates, bisphenols, biocides, UV stabilizers and flame retardants [58]. Additives may migrate via the plastic life cycle to become particles; these then become ‘free’ chemicals once leached out of the plastic [58]. These distinct emissions may have diverse environmental fates [59]. Moreover, the potential for additives to adsorb to, and/or re-adsorb onto plastic particles further complicates their environmental behavior and may increase the risk of long-range transport across global regions [60].

#### Microbiological pathways: plastisphere formation and microbial transport

Plastics in the environment provide a distinct ecological niche for microorganisms, forming a new ecosystem with plastics as the matrix, collectively termed the ‘plastisphere’ [6,61,62]. After plastics enter the environment, their surfaces create microhabitats that facilitate microbial attachment, persistence, interaction and dispersal [61,63]. Microbial communities assembled in plastispheres have been widely reported to exhibit community structures and functional profiles that differ from those observed in natural habitats, with their assembly shaped by polymer properties, additives, surface weathering, ambient environmental conditions and source microbial communities [49,64–66]. Growing evidence shows that pathogens and antimicrobial-resistant microbes are often enriched in plastispheres, threatening the health of humans and other organisms [21,23,67,68]. Because plastics are persistent and mobile substrates, they may further facilitate the dispersal of pathogens, antibiotic-resistance genes, viruses and other bioactive agents across aquatic, terrestrial and atmospheric compartments [6,63]. Beyond these health-related considerations, plastisphere communities may also modify ecosystem processes, including biogeochemical cycling [21,49,69]; these Earth-system implications are discussed in the section entitled ‘Biogeochemical cycling impacts’.

### Human health impacts

Micro- and nanoplastics have been detected in human tissues and biological samples, including blood, lungs, placenta and arterial plaques [70]. These findings have raised concerns about human exposure to plastic particles and their associated chemicals. Toxic chemicals used in plastics can disrupt immune and endocrine functions, cause cancer and lead to skin or eye irritation [71]. A recent clinical study reported microplastics or smaller nanoparticles in carotid artery plaques from ∼60% of >200 surgical patients [15]. During ∼34 months of follow-up, patients with detectable particles had a higher incidence of myocardial infarction, stroke or death than those without detectable particles [15]. Experimental studies further suggest biological plausibility, showing that microplastic exposure can induce intestinal inflammation, reduce sperm counts and impair reproductive outcomes in mice [72,73].

Breathing air and ingesting food and drink are the major plastic exposure pathways for humans [12]. The total exposure level is referred to as the ‘plastics exposome’. Compared with rural areas, urban air usually has more additives and plastics, because of more and larger emission sources, highlighting the need to prioritize governance of the urban plastics exposome [74,75]. A survey conducted in the USA shows that people drinking bottled water may consume 90  000 particles annually, while those drinking tap water take in 4000 particles [76]. These ingestion estimates may be underestimates because microplastic information is unavailable for most foods, including poultry, beef, vegetables and grains.

Indoor environments play an important role in human exposure, and more attention to indoor environments is needed to better characterize total environmental plastics leakage. For instance, data from a few indoor point sources, like releases from indoor dryers and furnishings, show that indoor air pollution by plastics is higher by one order of magnitude or more when compared with outside air; this estimate could be even higher if every source were known [77]. Diet is another important source of exposure to additives and plastics; however, a lack of information about many foods has hindered dietary exposure evaluations [14]. Rice, the staple food for >50% of the global population, contains as much plastic as many seafood products [78].

These findings highlight the need to better determine how plastics move from environmental sources to food, drinking water, air and ultimately the human body. Overall, although evidence for human exposure and biological effects is growing, stronger studies are still needed to connect exposure pathways, internal doses, biological mechanisms and long-term health outcomes in humans.

### Biogeochemical cycling impacts

After entering the environment, plastics can influence elemental biogeochemical cycling through their particle properties, chemical leachates and plastisphere communities. We next discuss how plastic pollution affects carbon and nitrogen cycling, and the underlying mechanisms, drawing on representative examples from different environmental systems.

#### The effect of plastics on carbon biogeochemistry

Microplastics influence carbon cycling through changes in the physical and chemical environments that govern organic matter storage and transformation in soils and sediments [22,51]. Once incorporated into soils, plastics have been shown to alter soil properties, such as aggregation, pH, bulk density and porosity [79,80]. This influences the distribution, persistence and potential loss of soil organic carbon [81]. Microplastics may facilitate the mineralization of soil organic matter by modifying microenvironments for oxygen availability, dissolved organic carbon (DOC) concentrations and electron transport capability [82]. In addition, plastics, particularly polystyrene and polyethylene, are composed predominantly of carbon (up to 90%); their slow decomposition and incorporation into soil aggregates can increase soil carbon concentrations, raising important questions about their contribution to soil carbon storage. Consequently, updated methodologies are needed to accurately quantify soil carbon stocks [83].

In addition to physicochemical effects, plastics also reshape carbon cycling through direct interactions with soil microbial communities. This influences microbial diversity, composition and metabolic activity [49,62]. Both plastic polymers and plastic-derived DOC can be utilized by microbes as carbon sources [49,51,84]. Experimental evidence shows that a 1% presence of conventional polystyrene microplastics can reduce carbon fixation by up to 18.7%–32.3%, by directly interfering with autotrophic microbial carbon fixation and indirectly altering soil organic carbon availability [85]. Moreover, plastics degradation under solar radiation reshapes the amount and composition of DOC in aquatic ecosystems [86,87]. In parallel, virus–host interactions in the soil plastisphere also alter microbial resource allocation and metabolism through changes in carbon use efficiency [88].

Although plastics contribute ∼4.5% of global greenhouse gas (GHG) emissions [89], their environmental effects on methane (CH_4_) emissions vary with particle type, ecosystem and soil conditions. Low-density polyethylene, for example, produced 4100 pmol g^−1^ d^−1^ of CH_4_ when exposed to ambient solar radiation in seawater [90]. In estuarine and coastal wetlands, microplastics increase CH_4_ emissions, with biodegradable microplastics having a stronger effect than conventional microplastics [91]. In contrast, in wastewater treatment ecosystems, the presence of polyethylene microplastics (10 mg L^−1^) inhibited acetogenesis and hydrogenotrophic methanogenesis, resulting in a 30.71% reduction in CH_4_ production during anaerobic digestion [92]. Similarly, prolonged exposure to polyvinyl chloride (PVC) microplastics at concentrations of 15–150 microplastic particles per liter significantly decreased CH_4_ production, by 11.0%–32.3%, in anaerobic granular-sludge systems [93]. Polystyrene nanoplastics also reshaped anaerobic granular sludge, decreasing methane production by 19.0%–28.6%, along with volatile fatty acid accumulation [94]. In rice paddies, PVC microplastics were found to induce more CH_4_ emissions than polypropylene (PP) and polyethylene microplastics [95]. However, another study found that polyethylene microplastics significantly reduced CH_4_ emissions, by 16.9%, from acidic paddy soil (pH = 5.0) and by 16.1% for alkaline paddy soil (pH = 8.2), while no effects were seen in neutral paddy soils [96]. Nanosized plastics have distinct impacts; for example, polyethylene nanoparticles increased DOC content and CH_4_ production by up to 1.8-fold and 10.1-fold, respectively, in rice paddies [97].

Taken together, plastics influence carbon cycling through three linked routes: physical alteration of carbon-storage environments, chemical inputs from polymer carbon and plastic-derived DOC, and biological changes in microbial carbon fixation, carbon use and methane metabolism. Variation among studies largely reflects which route dominates under specific polymer, aging, particle-size, redox and matrix conditions.

#### The effect of plastics on nitrogen biogeochemistry

Plastics in the environment may affect the pH, aggregate structure and other physicochemical properties of soil or sediment; this may affect the nitrogen cycle due to the physical and chemical property changes caused by plastic aging [98]. Plastics undergo changes in their physical and chemical properties over time, due to factors such as physical pressure, UV radiation, temperature and salinity, oxidative conditions and microbial degradation. This leads to decomposition in the natural environment [99]. The aging process varies depending on differences in plastic polymer type, concentration and environment; this leads to differences in the impact on nitrogen cycling. Physically, plastics gradually fragment, reducing particle size and increasing specific surface area [100]. This alters nutrient retention capacity and the pore structure of soil or sediment. Chemically, plastic-derived DOC can serve as a substrate for microorganisms, while additives leached from aging plastics can affect microbial activity and the soil metabolome [101]. This ultimately affects the nitrogen cycle as plastics decompose [102]. Varying concentrations of microplastics result in different aging-induced changes to their physical and chemical properties, leading to diverse effects on the nitrogen cycle [103].

Microplastics influence nitrogen primarily by affecting the composition and activity of nitrogen-fixing microorganisms. They achieve this by adsorbing and retaining inorganic nitrogen and altering the soil or sediment carbon-to-nitrogen (C/N) ratio [104]. For example, polyethylene reduces dissolved organic nitrogen while increasing inorganic nitrogen. This alters the structure of nitrogen-fixing bacterial communities and inhibits the activity of nitrogen-fixing microorganisms. However, Zhang *et al*. [105] found that the addition of microplastics increased the relative abundance of genes involved in nitrogen fixation and organic nitrogen conversion, possibly driven by the rapid release of DOC from biodegradable plastics. This increased the C/N ratio, enabling microorganisms to alleviate the nitrogen limitation through increased nitrogen fixation.

For nitrification and denitrification, microplastics mainly affect the nitrifiers and denitrifiers by changing soil or sediment aeration, releasing DOC and plastic additives [106,107]. Huang *et al*. [107] found a positive correlation between DOC levels and nitrification and denitrification rates. The plastic additives released during the aging and degradation of microplastics can be toxic to nitrifying and denitrifying microorganisms and inhibit nitrification and denitrification, although the stimulatory effect associated with plastic-derived DOC release appeared to outweigh additive-related inhibition under the conditions examined [108,109]. However, this balance is not universal, and additive toxicity may dominate at high exposure concentrations or for polymers releasing substantial amounts of inhibitory compounds. A meta-analysis study by Su *et al*. [69] concluded that microplastics led to a 140.4% increase in N_2_O emissions and only a 4.9% increase in nitrification rate; the denitrification rate increased by 17.8%, while the abundance of genes associated with denitrification increased by 10.6%.

Few studies have investigated the effects of microplastics on other nitrogen cycling processes, such as anaerobic ammonia oxidation [110–115], nitrifier denitrification [116] and fungal and chemical denitrification [117]. However, Hong *et al*. [118] showed that the response of anammox granular sludge to polyethylene terephthalate (PET) was concentration dependent. Exposure to 0.1–0.2 g L^–1^ PET had no significant effect on anammox efficiency, whereas 1.0 g L^–1^ PET reduced anammox activity by 16.2%. This inhibition was associated with weakened granular-sludge structure, increased oxidative stress, and declines in anammox bacteria and genes involved in energy, cofactor and vitamin metabolism. Another study showed that PVC microplastics inhibited anammox activity [119]. Su *et al*. [69] found that microplastics mainly increased N_2_O emissions, by promoting bacterial and fungal denitrification, but they did not increase chemical denitrification in estuarine ecosystems.

Collectively, plastics affect nitrogen cycling mainly by changing substrate supply, C/N balance, aeration, redox conditions and the functional microorganisms responsible for nitrogen transformations. Differences among studies therefore reflect how plastic aging, plastic-derived DOC supply, additive leaching, exposure concentration and matrix conditions shift the balance among nitrogen fixation, nitrification, denitrification, anammox and N₂O production.

Given the complexity and context dependence of plastic effects on biogeochemical cycling, future studies should integrate larger-scale and cross-system designs with comparable exposure metrics and biogeochemical endpoints to better resolve how plastic type, weathering status, associated chemicals and microorganisms, and environmental context jointly regulate carbon and nitrogen cycling.

### Plastic pollution and the triple planetary crises

The triple planetary crisis, comprising climate change, pollution and biodiversity loss, represents one of humanity’s most critical challenges, threatening planetary health and sustainable development [120]. Plastic pollution is increasingly recognized as a cross-cutting driver of the triple planetary crisis by amplifying its interconnected consequences [13].

As carbon-based materials, plastics contribute to GHG emissions across their life cycle. From the extraction and refining of hydrocarbons through polymer production, and on to product use and end-of-life disposal, each phase of the plastics’ life cycle releases GHGs such as CO_2_ and even CH_4_. The production of one metric ton of plastic resin generates ∼2 metric tons of CO_2_ emissions [13,121]. Plastics caused 4.5% of global GHG emissions in 2015, and may reach 15% of the global carbon budget by 2050 [89]. The majority of this carbon footprint arises from energy-intensive resin manufacturing and other production processes, but waste-management practices like incineration or open burning of plastic waste also contribute additional GHG emissions [13,121].

Plastics can exert climate effects indirectly after entering the environment, as mentioned above, as they gradually release their stored carbon into the environmental media along with weathering processes [5,51]. Besides, as both physical particles and chemical hotspots, plastics can alter the fundamental properties of environmental media (e.g. soil aggregate structure, porosity, pH and hydrology) [51], while changes in environmental properties have cascading effects on the (micro)biota [19,122]. All these modifications can disrupt biogeochemical cycling processes, and ultimately enhance emissions of GHGs, such as CO_2_, CH_4_ and N_2_O, contributing to climate warming [19,22,123,124]. Microplastics in soils and sediments can alter microbial activity and accelerate N_2_O emissions, with increases exceeding 140% under microplastic exposure [125]. Such responses indicate that microplastics can influence coupled carbon and nitrogen cycling, with potential consequences for long-term soil fertility and climate feedbacks [49].

Beyond climate impacts, plastic pollution also poses progressively greater risks to biodiversity via the impacts of its physical, chemical and microbiological dimensions [6,18,54,65,126]. Furthermore, plastics in the environment can interact with co-existing contaminants (e.g. heavy metals, polychlorinated biphenyls (PCBs) and antibiotics) and modify their bioavailability, environmental behavior and ecotoxicity, posing indirect health risks to organisms [127–130]. Plastic ingestion and accumulation have been documented across nearly all major taxonomic groups, with adverse effects ranging from physiological stress and disrupted feeding and reproduction to internal injury and death [54]. At the microbial scale, the plastisphere has emerged as a hotspot for antibiotic resistance genes and potential pathogens [23,67]. The enrichment of resistance traits and evidence of horizontal gene transfer suggest that plastic debris may facilitate the spread of antimicrobial resistance in both terrestrial and aquatic environments [23,67]. Therefore, plastic pollution may accelerate species decline across ecosystems.

## PLASTIC POLLUTION MITIGATION FROM A LIFE-CYCLE PERSPECTIVE

The demand for plastics continues to increase with growing waste and pollution, if not well managed. Regulators alongside researchers in industry and academia continue to search for approaches to control the corresponding pollution. Usually approaches imply systemic change to how plastic is used by society as the main issue to address, though life-cycle management and technological innovation can also play important roles [131]. Here, we present a hierarchical overview of solutions from most preferred to least preferred (Fig. 3).

**Figure 3.**
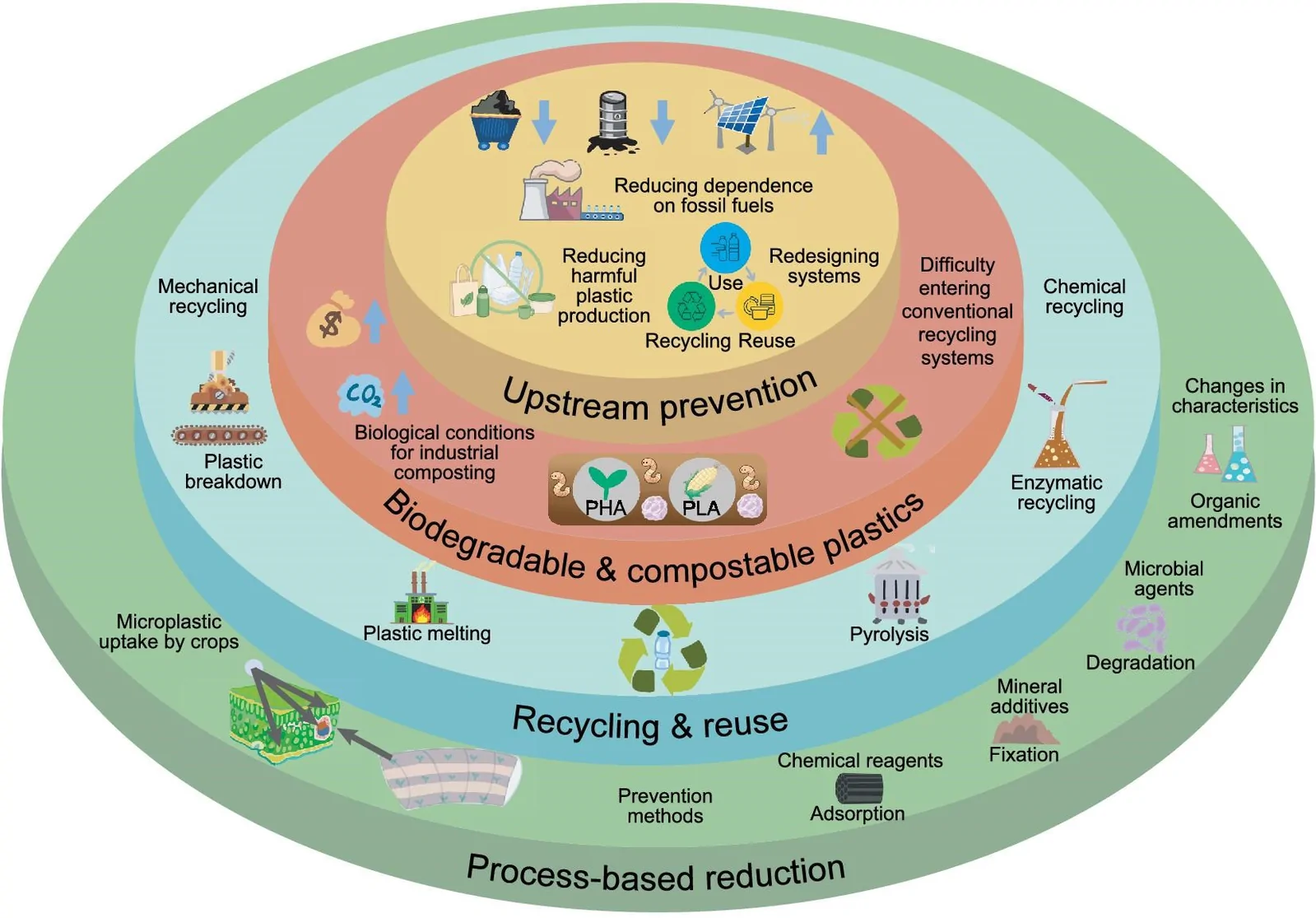
A hierarchical framework of plastic pollution mitigation. The innermost tier emphasizes upstream preventive measures, including reducing dependence on fossil fuels, optimizing system design and decreasing the production of harmful plastics. The second tier addresses the strategy to promote the development and application of biodegradable and compostable materials such as polyhy- droxyalkanoates (PHAs) and polylactic acid (PLA), as alternatives to conventional plastics. The third tier outlines recycling strategies, encompassing mechanical, chemical and biological routes for recirculating plastic materials. The outermost tier comprises four process-based strategies to reduce already existing microplastic contamination.

### Upstream prevention

The global plastic pollution crisis is fundamentally a crisis of overproduction and linear consumption [132]. Packaging accounted for roughly 40% of plastic use and ∼60% of global plastic waste generation in 2022, reflecting its predominantly single-use characteristics and short lifetime [2,133]. Upstream prevention includes strategies designed to tackle plastic pollution at its source, before materials ever become waste. This includes phasing out or banning non-essential, problematic and single-use plastics, redesigning products and packaging, material substitution, and implementing reuse and refill systems [134]. Thus, this approach is widely recognized as one of the most effective strategies by reducing the production of virgin, fossil-fuel-derived polymers and eliminating non-essential single-use items. Indeed, over 90% of plastic is produced using petroleum, so the plastic industry heavily depends on fossil fuels [26]. Considering the highly organized carbon and hydrogen structure of plastic waste, disposal or combustion pollutes the environment, wastes substantial resources and releases carbon as GHGs. Thus, upstream strategies are widely considered to have high potential to directly mitigate GHG emissions and prevent the generation of unmanageable microplastics. While international frameworks increasingly advocate for upstream interventions, establishing binding global production quotas or caps on virgin plastics has proven exceedingly difficult to implement and enforce [135]. Furthermore, the petrochemical industry’s massive sunk costs in extraction and manufacturing infrastructure create a so-called carbon lock-in, incentivizing continued overproduction and driving intense resistance against systemic production reductions. Consequently, achieving meaningful upstream prevention requires overcoming these entrenched economic barriers and securing unprecedented international regulatory coordination.

### Biobased, biodegradable and compostable plastics

Biobased, biodegradable and compostable plastics refer to various technologies and plastic life cycles. Biobased means the raw monomers and materials to make the plastic are of biological origin. Some of the high commodity plastics, like polyethylene, can be biobased. Biobased plastics do not inherently mean they are biodegradable or compostable, but this is often confused with terminology like ‘bioplastics’. Biodegradable plastics can biodegrade under certain conditions at their end of life, whereas compostable plastics are a subset of these that only biodegrade at the conditions of an industrial composter. Biodegradable plastics and compostable plastics may be made using fossil fuels, and are not necessarily biobased. Confusion about these terms has led to initiatives like the European Union’s policy to prevent ‘greenwashing’ through the proposed ‘Green claims’ directive, by ensuring that the biodegradation conditions are clearly labelled on plastic materials claiming to be biodegradable [136]. For consideration of the circular economy, it should be clearly differentiated that bioplastics could play a role, but biodegradable/compostable plastics should be considered as a single-use stream and therefore an option when circular economy solutions are not feasible (such as for soil mulching).

There are several major concerns with biodegradable plastics. The first is related to the greenwashing claim that they may not degrade in environments for which they were not tested (e.g. some plastics that degrade in soil may not degrade in marine water). Second, they can degrade into a variety of unknown transformation products, some of which may be toxic [137]. Third, much like conventional plastics, biodegradable plastics contain various chemical additives that can leach out and exert their own toxic effects on the surrounding environment. Biodegradable and compostable plastics are being produced in increasing amounts, which may reach 5 million tons in 2025 [138]. Currently, polyhydroxyalkanoates (PHAs) and polylactic acid (PLA) are two major categories. However, they only account for ∼1% of the annual production of over 400 million tons of plastic. Because biodegradable plastics are not directly reusable or recyclable, they are not considered part of the circular economy and should be clearly separated.

Assessing whether an alternative material is genuinely safer than conventional plastics therefore requires going beyond generic labels such as ‘biobased’ or ‘biodegradable’. At minimum, polymers and products should be evaluated using criteria that include: (i) the presence and hazard profile of additives and residual monomers; (ii) degradation behavior and persistence in relevant receiving environments (soil, freshwater, marine, industrial and home composting); (iii) the identity and toxicity of degradation and transformation products; and (iv) realistic exposure pathways for humans and ecosystems across the life cycle [139,140]. In this framework, polymers such as PVC and PP are not assumed to have similar environmental and health burdens simply because they are both ‘conventional plastics’. They are instead distinguished by their additive packages, their propensity to form micro- and nanoplastics, their release of hazardous substances during use and at end of life, and the feasibility of their safe collection and recycling. The same logic applies to biobased and biodegradable alternatives. Their net benefit relative to conventional plastics depends on a multidimensional assessment of hazard, degradation behavior and exposure, rather than on feedstock origin or compostability claims alone [137,141].

### Recycling and its barriers

Transitioning from a linear to a circular economy has been widely promoted as a promising strategy to mitigate global plastic pollution [142]. Despite significant initial investments in plastic recycling initiatives, only ∼9% of plastic waste was recycled globally [2]. This stark statistic raises the critical question: why has recycling failed to provide an effective solution for plastic waste management? Plastic recycling technologies can be broadly grouped into mechanical recycling, solvent-assisted purification, thermochemical conversion, chemical depolymerization, enzymatic recycling and integrated or hybrid systems. Plastic recycling technologies, barriers and environmental trade-offs are summarized in Table 1.

**Table 1.** Plastic recycling technologies, barriers and environmental trade-offs.

Recycling technologies	Potential benefits for plastic pollution	Key technical and economic barriers	Environmental trade-offs
Mechanical recycling	Reduces demand for virgin resin and can lower life-cycle impacts when high-quality, well-sorted streams are available; enables limited closed-loop applications [143,148].	Strongly dependent on accurate sorting and low contamination; chain scission and property loss limit recycling cycles; multilayer and composite plastics are difficult to treat [144,145].	Downcycling into low-value, short-lived products; additives and contaminants remain in the loop; does not address microplastic generation during use and reprocessing [148].
Chemical recycling	Can treat streams unsuitable for mechanical recycling and recover monomers or feedstocks with high purity, including some thermosets [157,158].	High capital and energy costs; lower yields and complex operation; many technologies remain at pilot scale; halogenated plastics such as PVC and fluoropolymers are problematic [153, 154].	High energy use and GHG emissions; risk of shifting plastics to fuel use under a ‘recycling’ label; potential emissions of hazardous substances if control is weak [153, 154].
Solvent-assisted recycling	Enables high-purity recovery and retention of molecular weight; improves quality of recyclate from complex products; can remove some contaminants [151, 152].	Requires efficient solvent recovery and management; economics depend on solvent recycling and scale; polymer-specific solubility limits applicability [223–225].	Risk of volatile organic compound (VOC) emissions and solvent losses; hazardous additives can be extracted into solvent phase and require additional treatment [160,161].
Integrated/hybrid systems	Can increase overall recovery from mixed waste by directing each fraction to the most suitable technology; offers flexibility as technologies evolve [152].	Requires high investment, sophisticated sorting and coordination; optimization is complex and context-specific; strong data and governance needed [151, 160].	Residuals may still go to incineration or export; system complexity can obscure real environmental performance and enable ‘greenwashing’ if evaluation metrics are weak [160].

Mechanical recycling, the most prevalent approach, involves breaking, melting and remolding plastics either into the same plastic product [143] or as low-quality pellets of mixed chemical composition [144,145]. Recycling into low-quality pellets is currently the most popular method for recycling waste plastics [146]. Mechanical recycling requires careful classification and separation of plastic products, because most plastics are not single, pure polymers but complex materials that can include copolymers, additives, fillers and degradation products with a broad range of molecular weights. This chemical and structural complexity makes it difficult to achieve clean, well-defined polymer streams for high-quality recycling and also contributes to the diverse composition of micro- and nanoplastics observed in the environment [147]. Once separated, the plastics are shredded, cleaned and melted to produce new plastic. Mechanical recycling is sensitive to pollutants such as contaminants and additives [143–145]. The process shortens the polymer length, affecting the properties (such as toughness or hardness) and their ability to be processed into new materials [148]. Different types of plastics cannot be sufficiently blended after melting, and a small number of incorrect types can reduce the quality of an entire batch of plastics. This makes it important to carefully classify and sort plastics first, which is a social and logistical challenge that would involve designing plastics for sorting within the context of recently designed sorting infrastructure and the mechanical recycling industry.

Thermochemical recycling, such as gasification and pyrolysis, uses heat, catalysts or controlled atmospheres to break plastics down into smaller molecules. These intermediate products can then be distilled into naphtha, which is subsequently steam-cracked to yield monomers and other basic chemicals [149,150]. Pyrolysis can process mixed plastic wastes and products containing several layers of diverse plastics [151,152]. Chemical depolymerization provides another route by cleaving susceptible polymers into monomers or defined oligomers, which can then be purified and repolymerized to avoid chain shortening or quality degradation during mechanical recycling. This may facilitate the recycling of some thermosetting plastics, which are non-meltable and cannot be mechanically recycled. However, in practice, advanced recycling routes are often less economically efficient than mechanical recycling, with lower yields, higher GHG emissions or potential toxic emissions depending on the technology and feedstock [153]. Further limitations are that halogenated plastics, such as PVC and fluoropolymers, are incompatible with pyrolysis approaches because they release halogenated compounds during thermal treatment [154]. Enzymatic recycling is a biologically catalyzed form of depolymerization and may overcome some limitations for specific polymers, although it has not yet been demonstrated at scale for broad mixed-plastic waste streams [152,155]. To date, enzyme-based recovery has advanced most clearly for PET and some polyurethane systems, because these polymers contain hydrolysable ester or urethane linkages that are more accessible to enzymatic or chemical depolymerization than the inert carbon–carbon backbones of polyolefins such as polyethylene and PP [156–158].

Solvent-assisted recycling (SAR) offers a route for polymer recovery based on selective dissolution and purification, rather than thermal conversion or depolymerization [151,152]. Chemically compatible solvents enhance mass transfer by disrupting intermolecular interactions, suppressing crosslinking and enabling selective dissolution of target polymers [159]. This approach also enables cost-effective transport of dissolved polymers and facilitates impurity removal (e.g. food residues and multilayer composites) via gradient filtration. However, a concern may be the extraction of hazardous additives into the solvents, such as flame retardants, which may require additional separation and management [160]. As an example, polystyrene, particularly in expanded polystyrene (EPS), serves as a lightweight material for packaging, insulation (e.g. construction and refrigerated containers) and food service applications [152]. Recycling EPS poses challenges due to its low bulk density, necessitating energy-intensive thermal compaction methods (melting, hot air and infrared irradiation) that risk oxidative polymer degradation [161]. SAR offers an alternative approach. Spraying EPS with biodegradable limonene—a citrus-derived solvent used in cosmetics—dissolves the polymer matrix while avoiding chain scission. This process enables volume reduction (>90%) for economical transport, concurrent removal of contaminants (food residues and non-PS polymers) via filtration and direct composting of organic impurities. Comparative studies demonstrate limonene’s technical equivalence to conventional solvents (toluene and acetone) without associated toxicity concerns [161]. Nevertheless, solvent-based methodologies face intrinsic limitations in polymer specificity and operational scalability. For instance, PS recycling via dissolution-precipitation requires rigorous solvent/additive separation followed by pelletization, and overall process economics depend critically on solvent-recovery efficiency and waste-stream-management protocols [160].

A concise summary of development trends for these approaches is as follows:

- The exclusive reliance on a single pathway is diminishing, with an increasing number of projects adopting multistage, modularly integrated recycling strategies (for example, solvent-assisted separation first, followed by mechanical or chemical routes for further recycling).
- Advances in sorting and pretreatment (mechanical-recycling-based) technologies are needed and expected to improve the quality of recovered single-polymer streams and reduce downstream processing costs, if regulatory clarity is used to facilitate local sorting and logistics.
- Key challenges for chemical recycling include: reducing energy consumption, improving monomer purity and stability, reducing process complexity, strengthening environmental and safety management, and ensuring economic viability.
- Solvent recovery and management are critical determinants of the success of solvent-assisted routes. There is a need to develop more efficient solvent-recovery systems, approaches to minimize volatile organic compound emissions and separate extracted hazardous substances from the solvents, and robust regulatory and compliance pathways.

### End-of-pipe control of plastic degradation and pollution

Despite intensified global efforts in plastic waste management, only a limited portion of plastic waste undergoes effective recycling or controlled incineration, while ∼80% of it ultimately contaminates landfills or natural ecosystems [2]. Once in the environment, these macroplastics are subjected to continuous weathering through UV photodegradation, mechanical abrasion and biological processes, fragmenting them into increasingly smaller particles while releasing associated pollutants [16]. More critically, even with an immediate cessation of all plastic production, the ongoing degradation of existing plastic waste would continue to elevate microplastic concentrations in the environment [24,35]. This underscores the imperative to prioritize the mitigation of plastic pollutants to safeguard human health, wildlife and broader ecosystem integrity [5,24,33,162].

Effective mitigation necessitates the development and deployment of multiscale plastic and microplastic detection technologies, ranging from satellite imaging and Internet of Things sensor networks to track macroplastic accumulation, down to highly sensitive spectroscopic tools for quantifying microplastic contamination in complex environments [163]. Once identified, the final treatment and environmental remediation strategies diverge significantly based on the scale and accessibility of the targeted pollution. Macroplastic interventions primarily rely on physical extraction, utilizing mechanical river interceptors, aquatic collection booms and localized soil cleanups to physically remove the debris [164]. Addressing dispersed microplastic contamination, however, poses a profound technological hurdle. In aquatic pathways, advanced end-of-pipe infrastructure, such as membrane bioreactors, rapid sand filtration and electrocoagulation within wastewater treatment plants, can successfully intercept micro- and nanoplastics, though this often inadvertently transfers the concentrated pollutants into sewage sludge rather than destroying them [165]. For complex terrestrial matrices like soil, viable *in situ* remediation options remain critically limited; while emerging biological treatments leveraging plastic-degrading microbes and enzymatic pathways offer theoretical promise, practical large-scale treatments currently default to highly disruptive and economically prohibitive methods such as soil washing or thermal desorption [166].

Although local-scale debris cleanups from marine environments, coastlines and terrestrial environments are generally benign, the potential concern of introducing large-scale plastic removal technology infrastructure, particularly in ecologically sensitive areas, could lead to regrettable remediation by adding to the harm already caused by plastics. Plastic removal technologies in general are not considered a sustainable solution relative to upstream prevention [131]. Particularly in the context of the triple planetary crises above, any claims that plastic removal technologies are safe and sustainable need to be assessed independently, considering the climate costs, chemical emissions, organism mortality and biodiversity loss caused by the deployment of the technology, to prevent ‘greenwashing’ [131].

Incineration with energy recovery is often presented as a way to utilize the high calorific value of plastics and reduce the volume of residual waste, and life-cycle studies show that modern plants can contribute to energy supply when operated under strict emission controls [167,168]. At the same time, plastics are fossil-based materials, so burning plastic-rich waste streams is associated with substantial GHG emissions, and incomplete combustion can generate toxic by-products such as dioxins, acid gases and contaminated fly ash that require careful management [169,170]. Recent assessments therefore suggest that plastics-to-energy may have a limited, conditional role for truly non-recyclable fractions within stringent regulatory frameworks, but it should not be treated as a primary or risk-free solution to plastic pollution when climate and health impacts are taken into account [171,172].

## KEY BARRIERS AND FURTHER STEPS

Given the above-mentioned threats from plastic pollution, the fifth United Nations Environment Assembly (UNEA-5.2) requested the establishment of the Intergovernmental Negotiating Committee (INC) to develop a legally binding Global Plastics Treaty by 2024 [173,174]. The negotiation process was subsequently launched in 2022, when 175 nations agreed to forge a legally binding international treaty to end plastic pollution [1,12]. The committee held several meetings between 2022 and 2025, including INC-1 in Punta del Este, Uruguay; INC-2 in Paris, France (June 2023); INC-3 in Nairobi, Kenya (November 2023); INC-4 in Ottawa, Canada (April 2024); and INC-5.1 in Busan, South Korea (November–December 2024). However, after multiple rounds of INC meetings, negotiations for the plastics treaty collapsed at the Geneva meeting in August 2025 [175,176]. The plastics treaty failed to reach an agreement due to various key challenges. Here, we analyze the draft treaty text and relevant documents to summarize the main contributing factors as follows.

### Debate over the chemical inventory associated with plastics

Plastics play an indispensable role in modern society, and their economic value cannot be ignored [177]. However, various toxic chemicals contained in plastics pose significant risks to both human health and the environment, as they are continuously released throughout their entire life cycle from raw material extraction and manufacturing to use and disposal [17]. A substantial gap remains in clearly defining and targeting the specific polymers and additives that necessitate control measures [178]. Currently, comprehensive data and centralized databases are severely lacking across all stages of the plastic life cycle, including additives, chemical production, waste emissions and micro/nanoplastics. Scientists have compiled a database of over 16 000 ‘plastic chemicals’, including raw materials and additives such as stabilizers and colorants, of which at least 4200 are ‘persistent, bioaccumulative, mobile and/or toxic’ [17]. Furthermore, hazard data remain unavailable for over 10 000 chemicals, and there is no publicly accessible information detailing which plastics contain >9000 of these substances [17,50]. Despite existing national regulations and multilateral environmental agreements, >3600 plastic chemicals of concern remain unregulated globally [179].

To effectively identify key priorities for plastics chemicals within the treaty, the following critical strategies must be prioritized. First, the plastics treaty must establish a science-based ‘watchlist’ or ‘hazardous chemicals list’. This list would prioritize substances for phase-out or restriction based on internationally recognized criteria, considering their toxicity, persistence and potential for bioaccumulation. Chemicals of high concern should be immediately included, such as certain endocrine disruptors, carcinogens and persistent organic pollutants used in plastics [180]. Second, transparency must be mandated through comprehensive disclosure requirements. Producers should be obliged to disclose the full chemical composition of their plastic products and materials, enabling informed decision-making and accountability throughout the value chain. Finally, a ‘safe-by-design’ framework should be established to incentivize innovation and ensure that new plastic materials and products are developed without incorporating hazardous chemicals from the design and development stages, as well as design for a circular use of materials.

### Lack of plastics flow and pollution monitoring

The mantra, ‘*You can’t manage what you can’t measure*’ applies to plastic pollution. Robust information on global life-cycle plastic material flows is the foundation of plastics control, particularly information on plastics end-of-life disposal, including recycling, incineration, landfilling, mismanaged waste and leakage into the environment. Geyer *et al*. [2] presented the first global material flow analysis (MFA) of plastics ever manufactured from 1950 to 2015, while Houssini *et al*. [133] provided a global trade-linked MFA of plastics. Although several studies have tried to quantify global plastic material flows, they have adopted substantially varied scopes, methodologies and underlying assumptions. This has resulted in significant variability and uncertainty in quantifying plastic inflows, stocks and waste generation [181]. A related example is the estimation of human microplastic ingestion, where different exposure models and input data have produced highly divergent results. Early syntheses have been widely popularized in the media as suggesting that humans ingest the equivalent of a ‘credit card’ of plastic per week [182], but subsequent assessments have questioned these figures and highlighted substantial uncertainty in the underlying data and modeling approaches [183]. The current methodology for tracing global plastic material flows still lacks officially recognized protocols, consistent observational techniques, globally consistent data sources and high-resolution assessments [184]. Without a commonly agreed-upon understanding of global plastic flows, it is impossible to develop and implement effective interventions to regulate them within the plastics treaty. This necessitates high-resolution MFA to comprehensively track all stages of plastic pollution, encompassing fossil raw material extraction, resin production, product manufacturing, consumption, waste disposal [177] and cross-border transfers [185]. Therefore, the plastics treaty should account not only for a country’s current annual levels of plastic production and consumption but also for its historical cumulative plastic waste generation, national capacities for waste management, recycling and reuse, and the environmental impacts of its exported plastic products.

Moreover, the capacity to relate specific plastic flows with pollution and ecological damage hotspots is critical but constrained by the absence of robust monitoring systems [24,126,186]. Because countries and regions differ widely in their industrial structures and consumption patterns, their plastic material flows also vary substantially, leading to significant disparities in plastic leakage volumes and the characteristics of resulting microplastic pollution [187]. Uncertainties in data sources, quantitative methodological approaches and research assumptions across studies have produced inconsistent estimates of plastic losses and their ecological impacts [181]. It is crucial to develop robust monitoring to empirically correlate plastic material flow data with actual environmental pollution and damage conditions, especially plastic waste and microplastics in marine and terrestrial waters, such as the platform Integrated Marine Debris Observing System (IMDOS) [188]. However, global and national-level data on the current state of plastic pollution and historical plastic accumulation are extremely scarce [188,189]. Furthermore, the complexity of international trade networks makes source tracing and accountability exceptionally difficult, preventing a clear understanding of the full picture and origins of plastic material flows and pollution. Additionally, while measuring the carbon emissions of plastics throughout their entire life cycle is important, existing comprehensive methodologies are lacking [190]. This makes it difficult to establish a fair and universally accepted policy framework for defining plastic emission responsibilities, similar to the challenges seen in climate change negotiations. These dual deficiencies in scientific evidence severely hinder the international community from developing effective measures with clearly defined objectives and responsibilities, ultimately impeding the adoption of a universally accepted plastics treaty.

### Social costs of plastic pollution remain unclear

A clear calculation of the social costs of carbon emissions is essential for climate change governance [191,192]. Similarly, the foundation for building an effective global plastic management system relies on a clear accounting of the social costs of plastics [27], that is, quantifying the specific damage they cause throughout their entire life cycle. This includes direct and long-term impacts on public health, ecosystem degradation, and losses to key industries such as fisheries, agriculture and tourism [164]. Plastic pollution is estimated to cause more than US$1.5 trillion in health-related economic losses each year [193]. However, existing damage assessment models have significant shortcomings and are not precise enough, failing to capture the complex pathways and integrated harms of plastic pollution across borders and ecosystems (such as from land to sea) and its accumulation within ecosystems. This ambiguity in scientific understanding directly leads to regulatory blind spots, preventing the establishment of effective early warning and intervention mechanisms.

Furthermore, the responsibilities and impacts of plastic pollution are distributed highly disproportionately on a global scale. Some countries, despite being major producers, maintain relatively limited domestic consumption and plastic waste leakage [194]. Conversely, many major consuming countries, though not the primary sources of production, face severe leakage and pollution pressures driven by their massive consumption. Most strikingly, the burden falls heaviest on ‘downstream’ recipients, such as numerous island nations [195]. While their contribution to global plastic consumption is negligible, their unique geographical and environmental vulnerabilities force them to bear pollution consequences that far exceed their proportionate responsibility [195]. This mismatch between the nations that generate plastic pollution and those that suffer its impacts further exacerbates the inequitable distribution of responsibilities within the global plastics treaty negotiations. To address this disparity, comprehensive social cost-benefit assessment models are urgently required. Long-term damage models for climate change provide a successful example in quantifying long-term plastic pollution impacts to society. The integrated assessment model of plastics’ long-term damage can bridge this gap [177], which is a promising effort to address the unclear and unequal social costs of plastic pollution [196,197].

### Lack of effectiveness evaluation for plastic control measures

The current global landscape is characterized by a lack of robust plastic control measures and, equally critically, a lack of evidence that existing policies work [198]. It is highly challenging to effectively design a global treaty to end plastic pollution when there is limited evidence regarding which interventions are successful and which are not [199]. Currently, countries have adopted a wide variety of measures, ranging from bans on specific single-use plastic products and explorations of Extended Producer Responsibility (EPR) schemes to deposit-return systems and targets for increasing plastic recycling rates (Table 2). Many existing policies, while potentially effective in specific regional contexts, suffer from poor adaptability in other regions and can lead to significant environmental burden-shifting, such as exporting plastic waste, increasing emissions from alternative materials, shifting pollution from land to air through incineration and moving waste-management burdens to lower-income countries [200]. This issue is exacerbated by extensive global trade networks, which often result in pollution and waste-management responsibilities being merely transferred to other regions rather than being resolved at the source of waste generation [133]. Even widely recognized plastic policies, such as recycled content requirements, are not without their controversies and implementation gaps. This underscores an urgent need for independent scientific research to validate their net benefits and identify potential unintended consequences [131]. A major barrier to progress is the stark absence of comprehensive policy evaluation, which leaves decision-makers without clear evidence to assess the true costs, benefits and overall effectiveness of various measures, thereby perpetuating debates and hindering the adoption of the most impactful solutions.

**Table 2.** Selected recent plastic management policies and evaluations.

Target stage	Policy instruments	Regions/year	Policies	Comments (mechanism, evidence of effectiveness, barriers, burden shifting and transferability)
Production	Recycled plastics	Australia, 2021	National Plastics Plan: 50% average recycled content included in packaging [226]	Mandating recycled content stimulates demand for recyclates but relies on adequate sorting and reprocessing capacity; costs may be passed to consumers, yet the approach is highly transferable to other packaging sectors.
	Bag bans	China, 2021	The production of plastic shopping bags (<0.025 mm) is prohibited [227]	Banning thin bags effectively reduces visible litter but may shift consumption to thicker bags or non-woven alternatives; enforcement in informal retail remains a barrier, though the instrument is easily replicable.
	Single-use plastic products	Hong Kong, China, 2024	Ban on all single-use plastic containers, cups, cutlery and plates [228]	A comprehensive upstream ban directly curbs waste generation, yet it requires affordable, sustainable alternatives and consumer behavior change.
Consumption	Reuse	EU, 2025	Packaging and Packaging Waste Regulation 2025/40 (PPWR): 100% of intra-EU Member State transport packaging used for transport between companies must be reusable within a reuse system from 2030 [229]	Mandating reusable transport packaging promotes circularity but adds logistics, cleaning and hygiene costs.
Waste management	Recycling regulations	US, 2020	Increase the recycling rate to 50% by 2030 [230]	Provides a national benchmark for recycling.
	EPR	UK, 2025	Deposit-return system: all single-use drinks containers by 2027 [231]	Creating strong economic incentives for container recovery, with proven high return rates, but requiring upfront investment in reverse vending and retailer cooperation.
	Waste-management regulations	England, 2025	Government’s Simpler Recycling plans: workplaces with ≥10 employees mandated to arrange waste collection [232]	Improving segregation and recycling quality, yet it imposes compliance costs on small and medium-sized enterprises and depends on local waste contractor capacity.
	Taxes	EU, 2021	EU Plastic Tax: a charge of €0.80 per kilogram of non-recycled plastic packaging waste [233]	A fiscal disincentive internalizes environmental costs and encourages recycled content, but the tax may be regressive and could be passed to consumers; effectiveness relies on robust monitoring.
	Information instruments	Ocean Conservation Charity, 2021	Surfers Against Sewage—Million Mile Clean [234]	Raises public awareness and mobilizes citizen action, but their direct waste-reduction impact is modest and voluntary.
	Trade policy	Global, 2019	Basel Convention Amendments on Plastic Waste: Requires prior informed consent from contracting parties for most cross-border transfers of plastic waste [235]	The instrument’s effectiveness depends on customs capacity, monitoring and alignment with domestic waste-management policies to avoid shifting burdens onto countries with the least infrastructure.

### Significant innovation and application gaps for emerging technologies

Recent technological advancements in plastics have offered solutions to the plastic crisis, including novel alternative materials and revolutionary recycling processes, such as enzyme-catalyzed depolymerization, microwave-enhanced pyrolysis and metathesis-driven chemical recycling approaches (Fig. 4) [138,201,202]. While these emerging, pilot-scale plastic technologies present promising potential, their economic viability and environmental trade-offs remain inadequately assessed and poorly understood [203]. There is a significant lack of standardized metrics and comprehensive life-cycle assessments to verify whether these solutions can truly deliver on their promise at scale. Consequently, despite the continuous emergence of innovations in plastic substitutes and recycling technologies, most have not yet demonstrated a clear and measurable capacity to systematically replace conventional production, recycling and pollution control methods [204]. As a result, they fall short of fulfilling their anticipated role in mitigating the global plastic crisis.

**Figure 4.**
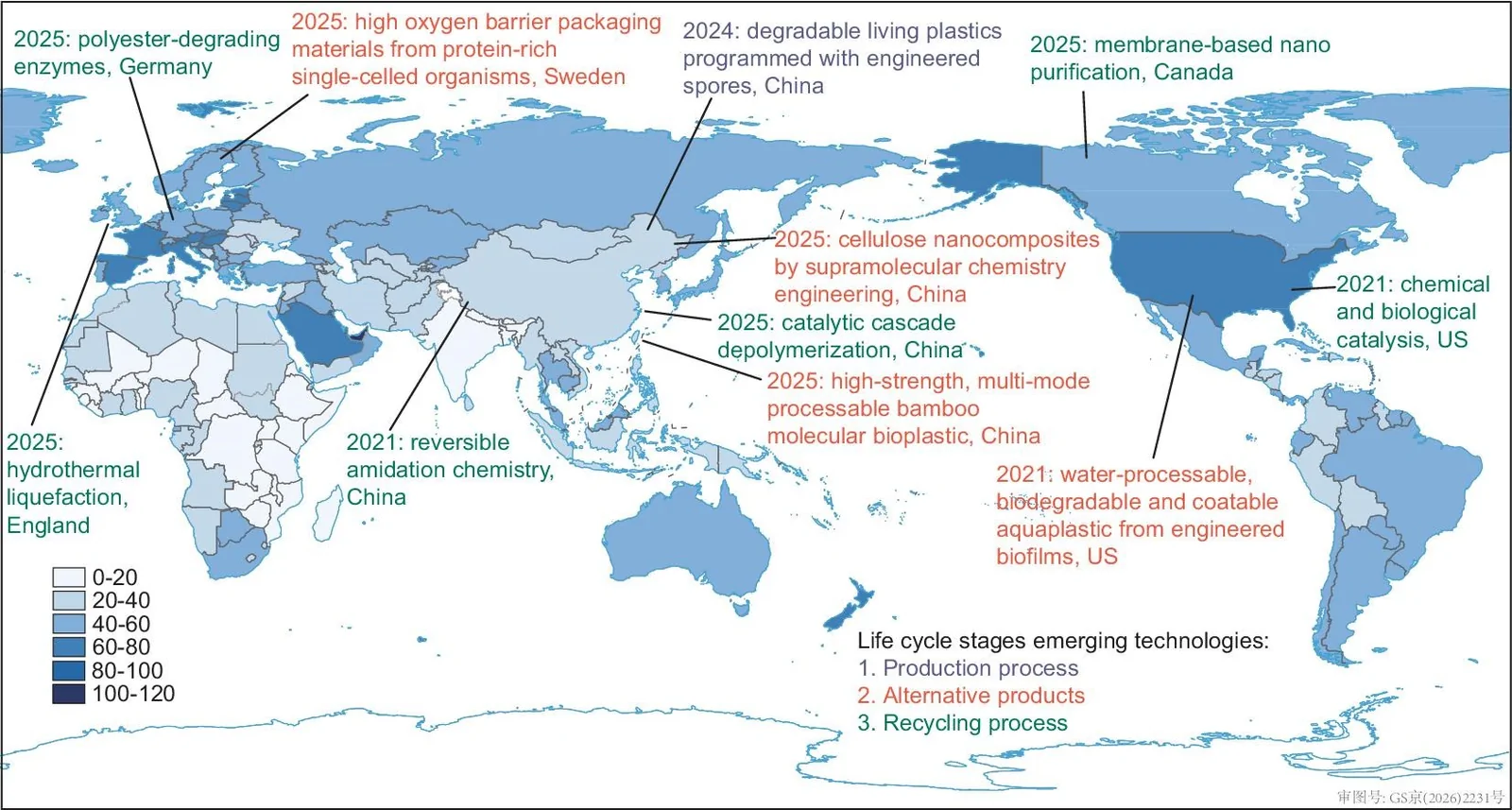
Estimated amount of plastic waste generated per person per year from municipal sources in 2020 (kg/capita) [164] and selected emerging technologies of plastic substitution and waste management [201,203,204,216–222]. Specific breakthrough technologies are highlighted across different regions, with the year of development indicated. The font colors correspond to the specific stage of the plastic life cycle these emerging technologies address: blue text indicates innovations in the production process; orange text represents the development of alternative products; and green text signifies advancements in the recycling process.

A critical barrier to scaling laboratory innovations into measurable real-world impact is their insufficient technical readiness for industrial deployment [140,205,206]. Key questions regarding their optimal application scope, adaptability to national contexts and the conditions needed to maximize their value remain largely unaddressed by comprehensive research [207]. Furthermore, the costs and investment requirements associated with new plastic technologies often lack transparency, creating a significant hurdle for commercialization. Addressing these challenges is therefore significant and demands a coordinated strategy among all stakeholders. This strategy must incorporate targeted research, strategically aligned investment and the development of evidence-based policy frameworks to effectively bridge the existing gaps. Considering the critical challenge of plastic pollution, the rapid scaling of innovative substitutes and recycling solutions urgently requires collaborative action from all relevant stakeholders, and therefore multi-stakeholder dialogue to address innovation and market readiness is readily encouraged.

### Priority actions to address policy implementation barriers

Global efforts to address plastic pollution include various policies, such as international treaties and policy initiatives, yet pollution levels remain persistently high. Overcoming this challenge will require comprehensive policy bundles that provide clear guidance on systemic transformation. This section focuses on policy priorities to overcome these barriers.

A strong science–policy–society interface to address global plastic pollution from a One Health perspective is needed. A fundamental solution lies in shifting from a linear economy (take–make–waste) to a circular economy, where each stage of the plastic life cycle minimizes plastic waste and plastic materials are continually returned into the economy [208,209]. Addressing this crisis requires a global framework capable of comprehensively managing the entire life cycle of plastics, including production, consumption, waste leakage, environmental pollution and planetary health consequences. Furthermore, it is essential to monitor and review plastic policy implementation annually to identify regulatory gaps and loopholes for moving forward. In response, the rational way forward is a comprehensive global plastic pollution governance framework, outlining specific priorities and actionable recommendations to guide coordinated efforts from all stakeholders. In this framework, the priorities and solutions to end plastic pollution include: (i) promoting sustainable production and consumption, (ii) minimizing plastic waste and leakage, and (iii) mitigating plastic pollution and damage, while safeguarding planetary health as an overarching goal.

#### Promoting sustainable production and consumption with policy support

To address the growing challenge of plastic pollution, a reduction strategy must be prioritized, phasing out problematic and unnecessary single-use plastic products. This can curb the production and consumption of plastics through measures such as plastic bans, taxes and fees, while accelerating the shift to sustainable business models. Science-based plastic phase-out criteria should be developed to identify high-priority plastics, applications and chemicals that require focused attention. As part of this strategy, there should be targeted bans on certain single-use plastic products, such as certain plastic bags, cutlery and straws, to reduce plastic waste at the source. In addition, concrete measures should be taken to develop global, science-based standards and lists of hazardous chemicals and problematic plastic products, and to establish clear phase-out timelines. These measures should include tightening chemical safety standards, mandating eco-labels that disclose chemical composition and encouraging a shift toward safer alternatives to conventional plastics, while explicitly avoiding regrettable substitution and acknowledging that many substitutes are not yet comprehensively assessed for their long-term environmental impacts [12].

Designing for plastic circularity requires supportive regulations. The regulatory framework should help businesses transition to alternative models that reduce plastic use [176]. Key design criteria include substituting hazardous additives and reformulating plastic and rubber compounds in products such as tires, vehicles, roads, paints and textiles, so that performance requirements can be met with lower toxicity and lower environmental release, while incorporating circular economy (CE) principles to extend product lifespan, support safe reuse, enhance repairability and increase recycling rates. However, product redesign often faces significant technical and economic hurdles, highlighting the need for a policy framework that encourages circular design and supports business models that align with it [210]. Reuse systems can operate in a closed-loop environment, and their benefits increase with scale, making collaboration among industries and governments at all levels crucial for reuse policies. Public incentives can play a key role in expanding these models, particularly through government financial support for necessary reuse infrastructure. Developing clear definitions of reuse and harmonizing related criteria can help establish an enforceable management framework for reuse systems. Promising reuse approaches include refillable beverage dispensers, subscription-based delivery systems and food container return systems with reverse logistics.

#### Accelerating regulations to minimize plastic waste and leakage

Enhancing plastic waste collection and recycling systems is important. A fundamental step in minimizing plastic waste and leakage is ensuring that waste is designed to be properly collected, sorted and managed. Key policy instruments to achieve this have been developed, including landfill and incineration taxes, EPR schemes for packaging and durables, deposit-return systems and pay-as-you-throw schemes [198]. Notably, deposit systems have proven highly effective in Europe, with an average return rate of 85% for covered products, which rises to 95% in Germany [211]. To further boost plastic recycling, it is critical to address market imbalances. Currently, the price of virgin plastics is often lower than that of recycled materials, a disparity exacerbated by fossil fuel subsidies. Creating a level playing field is essential for recycled plastics to compete with virgin plastics. However, recycled plastics are not inherently environmentally preferable under all conditions. Their environmental benefits depend on clean and well-sorted feedstocks, efficient recycling processes, high substitution rates for virgin polymers and sufficient material performance for the intended application. Under these conditions, especially for mechanical recycling, recycled plastics can reduce GHG emissions, energy use and water use relative to virgin polymer production [153]. The expansion of secondary plastics faces constraints from both existing recycling technologies and the availability of high-quality scrap. Challenges vary by waste stream and include limited recycling infrastructure for certain plastic types, the presence of hazardous additives and the complexity of dismantling products like electronic waste. Consequently, most recycling technologies are not yet economically viable without government subsidies [210]. Strategic investments in recycling technologies, combined with upstream design-for-recycling interventions, are required to secure viable feedstock.

A multifaceted approach is essential to prevent plastic from entering the environment. This involves establishing comprehensive life-cycle plastic waste collection and treatment systems, as well as addressing sea-based leakage sources such as abandoned fishing gear. To specifically tackle microplastic pollution, it is necessary to adopt upstream measures such as preventing industrial pellet loss, midstream eco-design innovations to reduce emissions and downstream solutions to capture microplastics already present in the environment.

#### Overcoming challenges of mitigating plastic pollution and damage

One of the most harmful forms of plastic pollution is microplastics originating from the abrasion of car tires, the shedding of synthetic textile fibres and the release of microbeads from cosmetic products, and they are estimated to account for 15%–31% of the plastics entering the oceans each year [212]. Many countries have started to ban microbeads in cosmetics by placing significant pressure on companies to eliminate them, while emerging technological solutions such as membrane bioreactors, photocatalysis and biodegradation processes offer promising pathways to address microplastic pollution [213]. In parallel, clean-up and remediation efforts, such as organized beach clean-ups and the installation of river litter booms, play a significant role in capturing plastic waste and mitigating associated environmental risks; though the use of this in sensitive ecosystems or as greenwashing to allow for pollution has been criticized [131].

A robust monitoring and evaluation framework, supported by globally standardized protocols and open-access data systems, is necessary. A transparent, data-driven decision-making framework is essential to assess the treaty’s effectiveness. The environmental impacts of plastic pollution extend far beyond visible waste. Microplastics have infiltrated terrestrial, aquatic and atmospheric systems, while associated toxic chemicals leach into ecosystems, posing significant risks to environmental and human health [6,57,60]. Monitoring these impacts is therefore critical for evaluating the success of mitigation measures and informing policy revisions [6,18]. A particular priority is assessing the bioaccumulation of hazardous substances and the effects of plastics on key ecosystem components, such as soil microbiota, which are fundamental to agricultural productivity and long-term food security [214]. To build a robust monitoring framework, three pillars are essential. First, globally standardized protocols are needed to track plastic pollution consistently across all environments, ensuring that worldwide data are comparable and reliable. Second, open-access data systems with public dashboards should be established to make monitoring information available to all stakeholders, enabling collaboration and informed data-driven decision-making. Third, a global digital hub under a recognized international body should be created to integrate and analyze data from the entire plastic life cycle. This hub should build on existing frameworks, such as the IMDOS [215], aligning with current efforts to improve management and prevent duplication. That being said, there will be a variety of monitoring methods and parameters, depending on the methodology used. As there is such a wide variety of plastic and microplastic pollution, these hubs and monitoring data should report all data alongside methodology, as monitoring plastics and microplastics is ultimately a method-dependent process.

## CONCLUSION

This Review synthesizes plastic pollution from understanding it to combatting it, presenting it as a multifaceted global crisis that links environmental behavior, physical–chemical–biological mechanisms of impact and life-cycle governance challenges. Plastic debris physically alters habitats and harms wildlife through ingestion and entanglement, while leaching toxic additives and persistent pollutants that contaminate ecosystems and threaten human health. Plastics also foster unique microbial communities that can harbor pathogens and disrupt biogeochemical cycles. Despite growing scientific understanding, critical knowledge gaps and technological challenges persist—from insufficient monitoring of plastic flows and inadequate regulation of thousands of plastic additives, to limited efficiency of current recycling and waste-management technologies.

Meanwhile, global policy responses remain fragmented and insufficient, reflecting a broader failure to enact effective international controls. Addressing this crisis, and reinvigorating the Global Plastics Treaty, requires a decisive pivot toward upstream interventions and systemic change. The Review calls for redesigning materials to be safe-by-design by eliminating hazardous additives, improving polymer degradability, reducing persistence, enhancing recyclability or preventing microplastic formation, embracing circular economy frameworks to minimize waste, and establishing robust global monitoring and accountability systems. Ultimately, tackling plastic pollution requires connecting scientific understanding with coordinated action, and integrating plastic pollution control into broader planetary health efforts alongside responses to climate change and biodiversity loss.
